# Vascular Variations and Incidental Pathologies in Potential Living Renal Donors Using 160-Slice Multidetector Computed Tomography Angiography

**DOI:** 10.7759/cureus.41502

**Published:** 2023-07-07

**Authors:** Sehrish Mehreen, Raja Rizwan Ahmed, Ruqaya Qureshi, Nadia Irfan

**Affiliations:** 1 Radiology, The Kidney Centre Post Graduate Training Institute (PGTI), Karachi, PAK; 2 Radiology, Memon Medical Institute Hospital, Karachi, PAK; 3 Urology, The Kidney Centre Post Graduate Training Institute (PGTI), Karachi, PAK; 4 Nephrology, The Kidney Centre Post Graduate Training Institute (PGTI), Karachi, PAK

**Keywords:** ct renal angiograms, renal transplantation, live related renal donors, renal vascular variations, renal arteries variations

## Abstract

Introduction: The aim of the study is to evaluate the vascular variations and incidental pathologies in potential living renal donors using 160-slice multidetector computed tomography (MDCT) angiography.

Methods: This is an observational study conducted at the Department of Radiology from January 2017 to May 2022. In this study, we performed retrospective data analysis of 61 CT renal angiograms, totaling 122 kidneys of potential renal donors, using a Toshiba 160 slice MDCT scanner with a four-phase CT image acquisition protocol, performed for pre-transplant workup. All patients had normal renal functions.

Results: Of our 61 patients, 34 (55.7%) were male and 27 (44.3%) were female, and their mean age was 31.2 ± 9.4 years. We have found 31 (50.8%) variations in the right renal arteries and 21 (34.4%) in the left renal arteries. Of these patients, 13 had bilateral renal arterial variations. The late confluence of the renal vein was found in 3.3% of males, multiple right renal veins in 7 (11.5%), and left renal veins in 2 (3.3%). By distributing the data according to gender, we noticed more diversity in the renal vessels of male patients. Left renal artery variations were more frequent in males (16, 76.2%) than in females (5, 23.8%), and they were statistically significant (p=0.02). Likewise, variations in the right renal arteries were also more frequently found in males (19, 61.3%) as compared to females (12, 38.7%). Right renal vein variations were more common in males (9, 81.8%) as compared to females (2, 18.2%) (p=0.05).

Conclusion: Frequent renal vascular variations and incidental pathologies in potential living donors were found by MDCT examination, and these vascular variations should be analyzed before renal transplant.

## Introduction

The global increase in the prevalence of end-stage renal disease (ESRD) confronts a great challenge for healthcare professionals. The ultimate treatment for ESRD is renal transplantation, apart from dialysis, which can greatly improve the quality of a patient’s life [1]. In developing countries, including Pakistan, renal transplantation has increased significantly, but very few studies have been conducted to assess renal vascular variations.

The most advantageous method of organ procurement is living kidney donation, which exhibits numerous benefits. To ensure the safety of the donor, every living donor should undergo a thorough medical assessment that includes a detailed medical history, examination, screening tests for blood and urine, an electrocardiogram, a chest radiograph, and an imaging workup for the evaluation of renal kidney anatomy and its vasculature [2].

Renal vasculature is renowned for exhibiting a broad range of variability. Knowledge of these patterns has gained significance not only for renal transplantation but also because it is essential to understand the anatomy of vessels prior to nephrectomy, vascular intervention for renal artery stenosis, and open surgery or endovascular stenting for abdominal aortic aneurysm [3-5].

The left kidney is usually favored for living donors because it is technically convenient to remove the left kidney due to the relatively long course of the renal vein [6]. However, a disadvantage of left donor nephrectomy is the frequent variations in renal veins, which include collateral venous pathways related to the left renal vein [7].

Renal artery diversities are divided into the extrarenal arteries, which include accessory and multiple renal arteries, and the early prehilar division. The accessory artery supplies the kidney, and it enters through the renal hilum [8]. Prehilar early division is a normal anatomical variant in which the main artery is divided within 1.5-2.0 cm from its aortic origin to the kidney [5,9].

Renal vein evaluation is divided into multiplicity, late confluence, and retroaortic (circumaortic). Renal vein multiplicity is identifiable as two or more renal veins; late confluence is termed when the venous tributaries join to form the renal vein at a distance of less than 1.5 cm with the inferior vena cava (IVC) [10,11]. The left retroaortic renal vein has a course posterior to the aorta before draining to the IVC. In the circumaortic renal vein, there is an accessory left renal vein that has a retroaortic course, while the other renal vein passes anterior to the aorta, constituting the circumaortic variant.

The non-invasive radiological investigations of donors include ultrasound color Doppler, multidetector computed tomography (MDCT), including CT renal angiography (CTA), and magnetic resonance imaging (MRI) or magnetic resonance angiography (MRA). Ultrasound is commonly used in living donor kidney evaluation for screening purposes in people with a family history of autosomal dominant polycystic kidney disease, for renal morphology assessment, and for any other anatomic variations [12].

The role of conventional angiography has significantly decreased due to the advancement of relatively less invasive cross-sectional imaging techniques. CTA is a less invasive modality that is usually well tolerated by the donors, and it gives comparatively more details concerning abdominal structures and vascular anatomy [9].

Our purpose is to provide an overview of possible renal vascular anomalies and variations and to assess other incidental findings that can have an impact on the selection of a renal donor.

## Materials and methods

The Institutional review board exemption was taken from the hospital Ethical Review Committee (ERC) for our retrospective observational study (ERC Reference No. 149-RAD-062022).

From January 2017 to May 2022, 122 living renal donors (including 34 males [55.7%] and 27 females [44.3%], whose mean age was 31.2 ± 9.4 years) were included in the study. In our hospital, "The Kidney Centre Post Graduate Training Institute," we are doing live-related renal transplants for which we need computed tomography (CT) contrast pyelograms and angiograms to see the vascular anatomy of donors, which will help in the anastomosis of vessels in renal transplant recipients at the time of transplant. After CTA, these patients are evaluated to determine whether they can be suitable donors or not for a renal transplant. For the procedure, all donors underwent preoperative intravenous contrast four-phase CTA before the planned nephrectomy, and the collected CTA images were analyzed for renal vascular variations and incidental pathologies. All renal donors were already enlisted in the renal transplantation program at our hospital.

The MDCT angiography protocol included four phases. Initially, the unenhanced phase was performed following a scanogram. This phase is excellent for assessing any calculus in the urinary tract or abnormal calcifications. Images were acquired from the dome of the diaphragm to the bifurcation of the common iliac arteries or the level of the iliac crest [13]. The second phase was performed with automated bolus tracking, in which 300 to 370 mg/mL intravenous nonionic contrast agent was given at an injection rate of 5 mL/s. The maximum contrast dosage was 120 mL, depending on the iodine concentration. The nephrographic phase was performed in the 100s, and it provides more information regarding renal parenchyma, especially for incidental small-sized renal masses [13,14]. This phase accurately illustrates the venous anatomy, particularly thin-caliber veins like the adrenal and gonadal veins [15]. Lastly, the pyelographic phase was acquired between 5 and 10 minutes after the initial intravenous contrast administration. The excretory phase demonstrates the accurate anatomy and pathologies related to the pelvicalyceal system and urinary tract. Concerning less radiation exposure, some institutes (not us) only take the scout image [16]. However, with advancement, it has been seen that the urothelial masses can easily be missed if only a scout image is acquired. A qualified radiologist reviewed the imaging data on a workstation with 2D and 3D reformats (vitrea 2). Discrepancies were resolved to serve as a gold standard. The reviewers studied each CT scan, including the axial images with multiplanar reformations (MPRs), maximum intensity projections (MIPs), and volume rendering wherever required. The reviewers used CT volume data to create three-dimensional angiographic and pyelographic images. Detailed renal vascular anatomy was evaluated through reformatted coronal and sagittal images and curved MPR. Volume rendering techniques were applied for three-dimensional CTA and were supported by MIP rendering techniques. The reviewers explained and classified the vascular anatomical details, like the number of renal arteries and veins on each side and the branching patterns; any vascular abnormality like stenosis or plaque; and incidental congenital and acquired renal abnormalities.

The data were entered and analyzed on IBM SPSS version 21 (IBM Corp., Armonk, NY). Cleaning and coding of the data were done before analysis. The mean ± STD was calculated for continuous variables like age, while the frequency with percentage was obtained for categorical data. The Chi-square test was applied to observe any association between variables. A p-value of ≤ 0.05 was considered significant.

## Results

We recruited 61 patients for our study, of whom 34 were male (55.7%) and 27 were female (44.3%). The average age was 31.2 ± 9.4 years, ranging from 17 to 52 years.

There were 21 (34.4%) patients who had variations in the left kidney arteries, while 31 (50.8%) had variations in the right kidney arteries. Among them, 8 (13.1%) had variations in only the left kidney arteries, 18 (29.5%) had isolated variations in the right kidney arteries, and 13 (12.3%) patients had bilateral variations (Table 1).

**Table 1:** Prevalence of renal vascular variations in donor of kidney transplant n= 61(%).

Renal vessels	Left only	Right only	Bilateral
Variation in kidney artery	8(13.1)	18(29.5)	13(21.3)
Variation in kidney vein	10(16.4)	4(6.6)	1(1.6)
Accessory renal artery	6(9.8)	8(13.1)	4(6.6)
Multiple renal arteries	6(9.8)	12(19.6)	0
Multiple renal vein	2(3.3)	7(11.5)	0
Renal artery prehilar branching	11(18)	10(16.4)	4(6.6)
Late confluence renal vein	2(3.3)	2(3.3)	1(1.6)

We found the late confluence of renal veins at 3.3% only in males with equal right and left distributions (two on each side). Multiple right renal veins were found in 7 (11.5%) and 2 (3.3%) on the left side.

When we distributed our data according to gender, we found that there was a male preponderance in the variation of kidney arteries. We noticed that left kidney artery variations were more common in males (16, 76.2%) as compared to females (5, 23.8%), and this association was statistically significant (p=0.020). Similarly, we observed that variated right kidney arteries were also more common in males (19, 61.3%) as compared to females (12, 38.7%), although this association did not have statistical significance (p=0.375) (Table 2).

**Table 2:** Association of gender with the variation in renal arteries, n(%).

Renal arteries	Male	Female	P value
All left kidney arteries	16(76.2)	5(23.8)	0.02
All right kidney arteries	19(61.3)	12(38.7)	0.375
Left accessory artery	4(66.7)	2(33.3)	0.57
Right accessory artery	5(62.5)	3(37.5)	0.68
Left multiple renal artery	4(66.7)	2(33.30	0.57
Right multiple renal artery	7(58.3)	5(41.7)	0.84
Left renal artery prehilar branching	8(72.7)	3(27.3)	0.21
Right renal artery prehilar branching	6(60)	4(40)	0.767

Similarly, variations in renal veins were also more frequent in males than in females. Right renal vein variations occurred more commonly in males (9, 81.8%) as compared to females (2, 18.2%) (p=0.05) (Table 3).

**Table 3:** Association of gender with variations in renal veins, n(%).

Renal veins	Male	Female	P-value
All left renal vein	2(40)	3(60)	0.46
All right renal vein	9(81.8)	2(18.2)	0.054
Left renal multiple veins	0	2(100)	0.107
Right renal multiple veins	6(85.7)	1(14.3)	0.09
Left late confluence renal vein	2(100)	0	0.2
Right late confluence renal vein	2(100)	0	0.2

**Figure 1:**
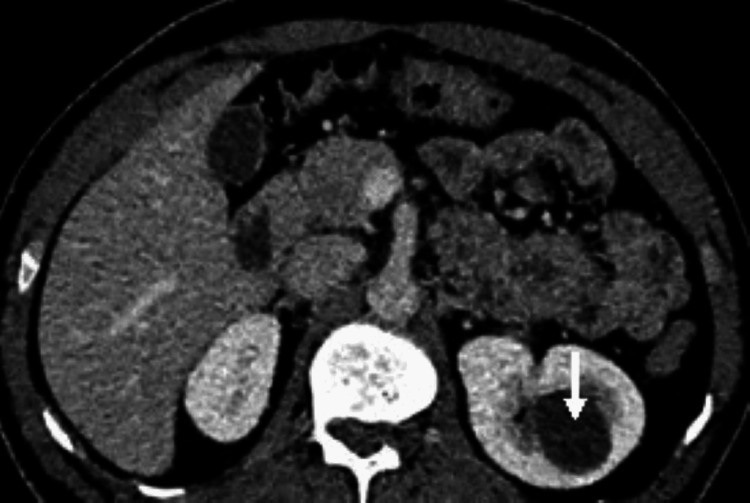
CT nephrographic phase, shows a cystic lesion at the upper pole corticomedullary junction.

**Figure 2:**
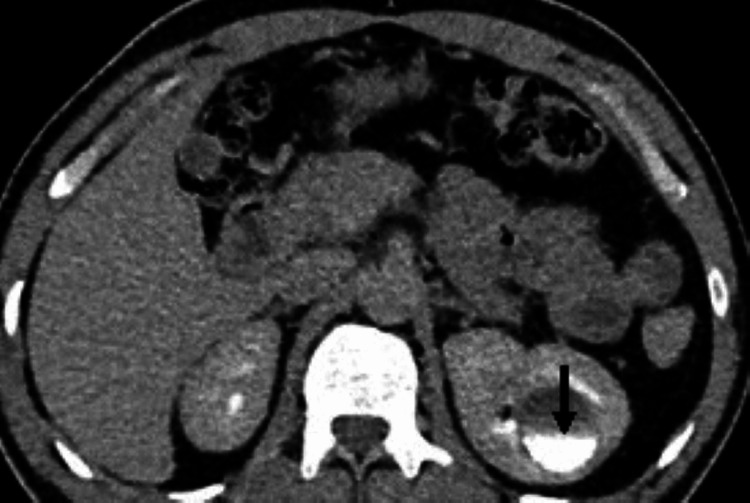
In the excretory phase, there is a progressive filling of the contrast in the lesion making fluid level, confirming that the cystic lesion is a calyceal diverticulum.

The incidental findings found in the donor’s kidneys were tiny (2-3 mm) calculi in two patients, a left renal calyceal diverticulum of 3.0 cm × 2.8 cm, which showed progressive filling of contrast on delayed phase (Figures 1-2), and one donor with pyramidal calcification diagnosed as medullary nephrocalcinosis.

Other incidental pathologies were cholelithiasis in two female patients, a right ovarian cyst of about 4.0 cm without any calcification or enhancing component in it, uterine fibroid in a donor measuring 2.4 cm × 2.7 cm, left inguinal hernia containing omental fat in one male donor, undescended right testis with mild hydrocele, and mild hepatosplenomegaly in one donor.

## Discussion

**Figure 3:**
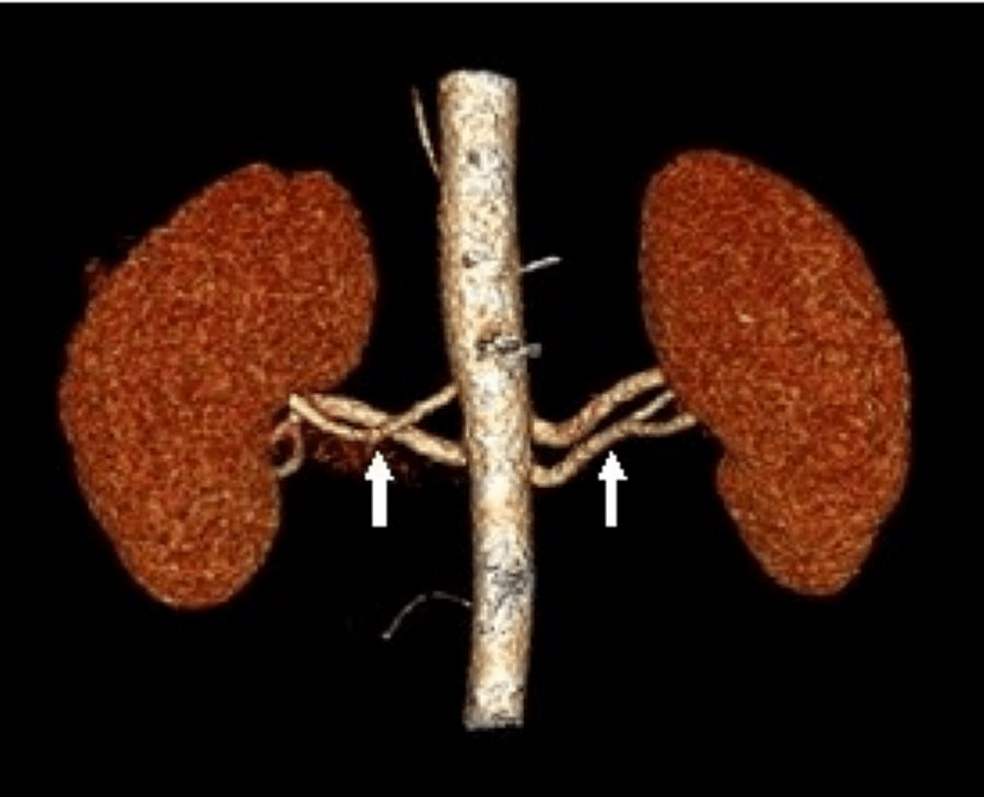
Multiplanar reconstruction image of CT renal angiography revealed bilateral accessory renal arteries separately originating from the aorta.

We report here five years of retrospective data from 61 renal donors with a total of 122 kidneys CTA and found profound and frequent vascular variations. Renal artery variations are common bilaterally, with a preponderance in the right renal artery (31, 50.8%) compared to the left renal artery (21, 34.4%). The prevalence of bilateral accessory renal arteries was 6.6% (four patients) (Figure 3 and Table 1), with only the right renal accessory artery at 13.1% (eight kidneys) and a 9.8% prevalence (six kidneys) of the left accessory renal artery.

Like other studies in the literature, we observed more accessory renal arteries on the right side. Ugurel et al. discovered a 42% incidence of accessory renal arteries on the right side in a previous study they conducted on 100 patients using a 16-detector MDCT scanner [16]. The majority of studies determining the prevalence of accessory renal arteries on MDCT include living renal donors, and most of the studies were based on a limited sample size.

**Figure 4:**
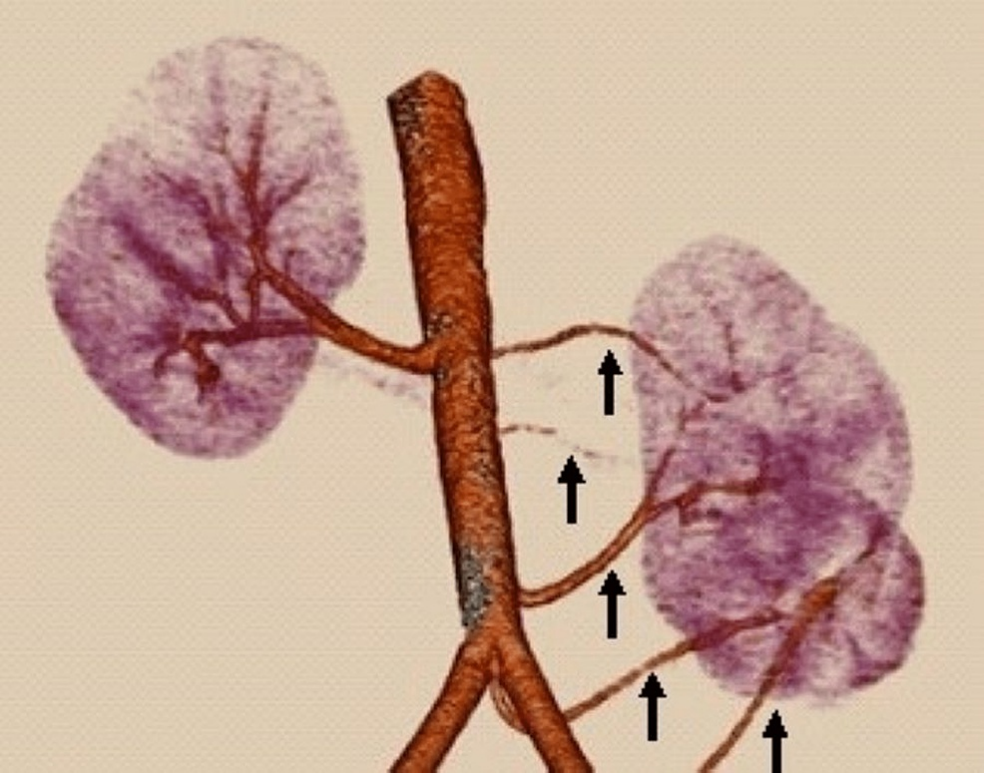
Multiplanar reconstruction image of CT angiography demonstrates five left renal arteries with separate aortic origins.

**Figure 5:**
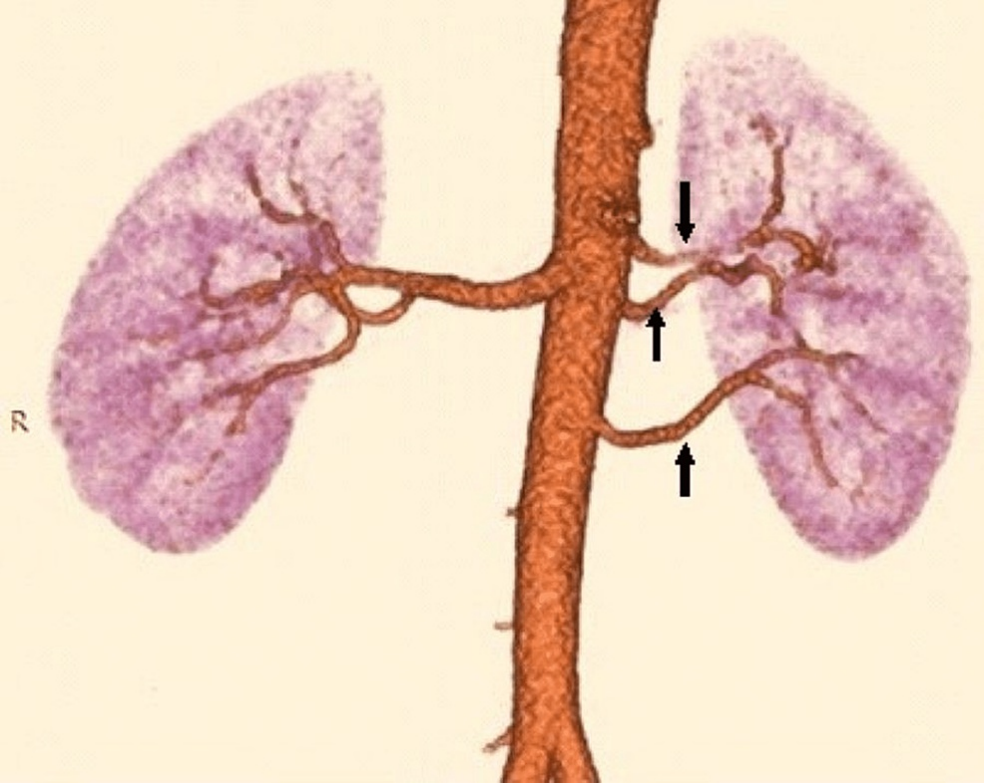
Multiplanar reconstruction image of CT angiogram of renal arteries showing three left renal arteries separately arising from the aorta and supplying the upper, mid, and lower poles, respectively.

Multiple renal arteries were also more frequently found in the right kidney, that is, 12 kidneys, with a prevalence of 19.6%, as compared to left renal arteries, which were seen in only six left kidneys with a prevalence of 9.8% (Figures 4-5). A study was conducted by Holden et al. with a limited number of donors, that is, 100 living renal donors for preoperative evaluation, and multiple renal arteries were found in 52 kidneys [17].

**Figure 6:**
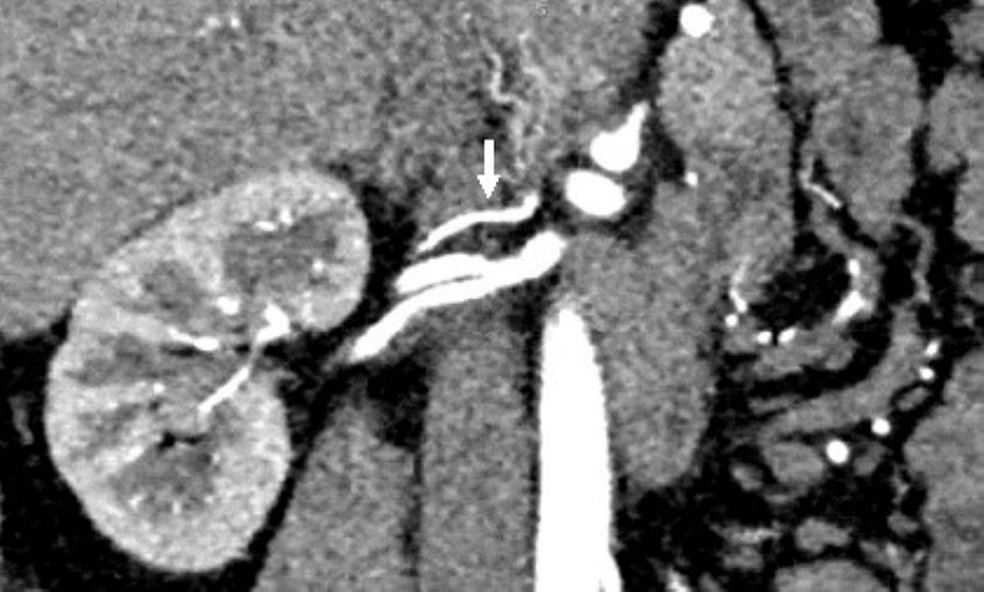
CT angiography shows prehilar early branching of right renal artery.

It is essential to recognize particularly early branching of the main renal artery (Figure 6), which divides 1.5 to 2.0 cm distally from its aortic origin. A literature review showed the prevalence of early renal artery division varies from 4.3% to 13% [17-19]. In our study, early branching of the main renal artery was found to be 18% on the left, 16.4% on the right, and 6.6% bilateral, which means there is a slight predominance of prehilar branching on the left side due to its short course towards the left kidney. According to a study conducted by Raman et al., the early division of the left renal arteries (less than 2 cm from aortic origin) was noticed in 21% of donors, while on the right side, the early branching was found in 15% of individuals [20].

Donor nephrectomy is not an absolute contraindication for renal vein anatomical variants [7,21]. However, variations such as retroaortic or circumaortic renal veins should be kept in mind before making an incision for nephrectomy. In our study, we have found one case of the left circumaortic artery in which an extra-renal vein was coursing behind the aorta. Many studies state that the retroaortic or circumaortic left renal vein may have an association with varicocele or pelvic congestion syndrome [21], but we did not find this association in our case.

**Figure 7:**
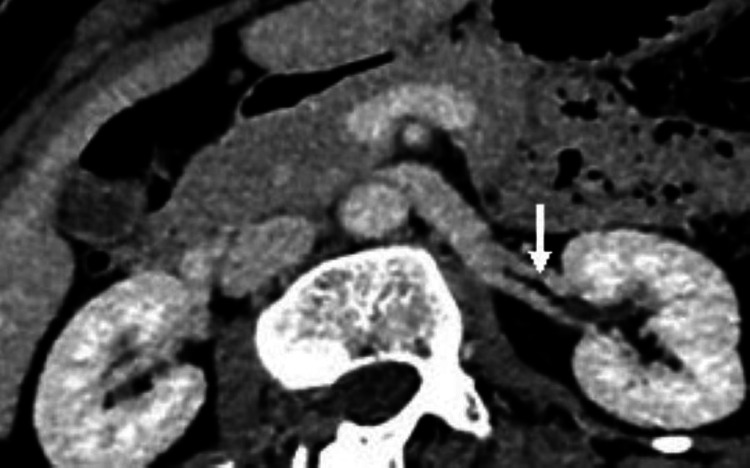
Nephrographic phase of CT angiography shows late confluence of left renal vein.

**Figure 8:**
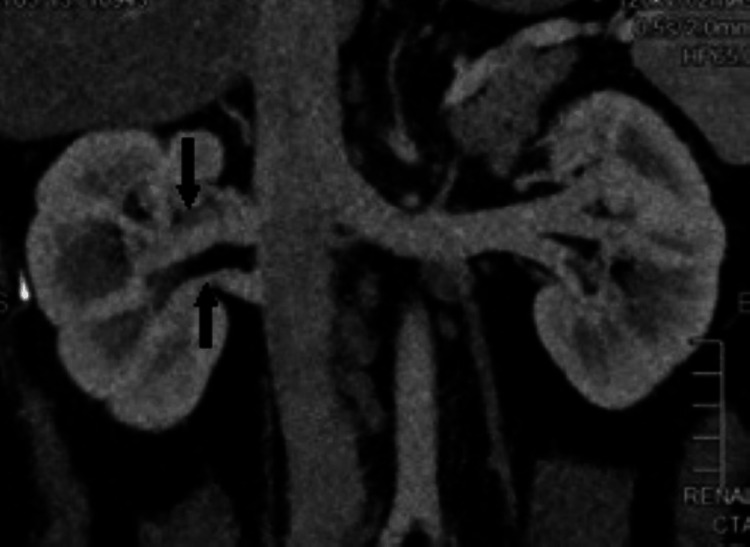
CT angiography image in nephrographic phase shows two right renal veins separately draining to the inferior vena cava.

In our study, the prevalence of late renal vein confluence was 3.3%, found only in males with equal right and left distribution (Figure 7) and two on each side. Isolated multiple right renal veins were found in 7 (11.5%) (Figure 8) and only 2 (3.3%) in left renal veins.

A limited number of previous studies investigated the association of renal vascular variations with gender. According to Kawamoto et al. [7] and Koc et al. [21], right-sided renal vein variations are more common in women. Apart from that, none of the studies showed an association between the prevalence of other renal vein variations and gender. We did not find any study that reported the association between renal artery anatomical variations and gender. In most parts of the world, cadaveric renal transplants are common, but in our country, Pakistan, and in our institute, we are dealing with live-related renal transplants, and there is a shortage of live-related donors because there are many circumstances where recipients cannot find suitable donors in their close family members and they have to be on dialysis. This is a major limitation, as we have limited transplants done in our institute.

## Conclusions

Renal transplantation remains a suitable and viable option for patients with ESRD. A thorough pre-transplant evaluation of the donor’s kidney is extremely important for both the donor and the recipient. Although there are several imaging methods available, currently CTA remains the preferred and gold-standard imaging technique despite the risks of radiation and nephrotoxicity.

Post-processing and 3D reformatting are crucial elements in renal donor evaluation and measurements. Our research found that different types of anatomical variations in renal arteries and veins are common and should not be underestimated. During the planning phase of a renal transplant or other renal interventional procedure, a CT examination of the renal vascular anatomy can greatly reduce the risk of operative complications.
